# Rapid Species Diagnosis for Invasive Candidiasis Using Mass Spectrometry

**DOI:** 10.1371/journal.pone.0008862

**Published:** 2010-01-25

**Authors:** Carine Marinach-Patrice, Arnaud Fekkar, Ralitsa Atanasova, Johanna Gomes, Laura Djamdjian, Jean-Yves Brossas, Isabelle Meyer, Pierre Buffet, Georges Snounou, Annick Datry, Christophe Hennequin, Jean-Louis Golmard, Dominique Mazier

**Affiliations:** 1 INSERM, U945, Paris, France; 2 Université Pierre et Marie Curie-Paris6, UMR S945, Paris, France; 3 AP-HP, Groupe hospitalier Pitié-Salpêtrière, Service Parasitologie-Mycologie, Paris, France; 4 Centre d'investigation Biomédical, Groupe hospitalier Pitié-Salpêtrière, Paris, France; 5 Laboratory of Molecular and Cellular Parasitology, Department of Microbiology, Faculty of Medicine, National University of Singapore, Singapore, Singapore; 6 AP-HP, Hôpital St Antoine, Service Parasitologie-Mycologie, Paris, France; 7 Université Pierre et Marie Curie-Paris6, ER4/EA 3974, Modélisation en Recherche Clinique, Paris, France; Duke University Medical Center, United States of America

## Abstract

**Background:**

Matrix-assisted laser desorption ionisation time of flight mass spectrometry (MALDI TOF-MS) allows the identification of most bacteria and an increasing number of fungi. The potential for the highest clinical benefit of such methods would be in severe acute infections that require prompt treatment adapted to the infecting species. Our objective was to determine whether yeasts could be identified directly from a positive blood culture, avoiding the 1–3 days subculture step currently required before any therapeutic adjustments can be made.

**Methodology/Principal Findings:**

Using human blood spiked with *Candida albicans* to simulate blood cultures, we optimized protocols to obtain MALDI TOF-MS fingerprints where signals from blood proteins are reduced. Simulated cultures elaborated using a set of 12 strains belonging to 6 different species were then tested. Quantifiable spectral differences in the 5000–7400 Da mass range allowed to discriminate between these species and to build a reference database. The validation of the method and the statistical approach to spectral analysis were conducted using individual simulated blood cultures of 36 additional strains (six for each species). Correct identification of the species of these strains was obtained.

**Conclusions/Significance:**

Direct MALDI TOF-MS analysis of aliquots from positive blood cultures allowed rapid and accurate identification of the main *Candida* species, thus obviating the need for sub-culturing on specific media. Subsequent to this proof-of-principle demonstration, the method can be extended to other clinically relevant yeast species, and applied to an adequate number of clinical samples in order to establish its potential to improve antimicrobial management of patients with fungemia.

## Introduction

Matrix-assisted laser desorption ionisation time of flight mass spectrometry (MALDI TOF-MS) has been recently described as an “ongoing revolution” because it allows rapid and accurate identification of bacteria [1], [2] and fungi [3], [4], [5]. Consequently, it is fast becoming an important and increasingly widely available tool in clinical microbiology laboratories. At present, accurate identification of genus/species of the pathogenic fungi is only performed on positive blood cultures grown from patient samples for 24/48. An additional 24/48 hours period, during which subcultures on specific media are grown, is required for the identification of the causative pathogen. Any advances that would shorten the time to correct diagnosis would be of some benefit to clinical management.

In this manuscript we addressed this issue for *Candida* species, which are one of the commonest etiological agents of invasive fungal infections as well as of nosocomial bloodstream infections [6]. Molecular-based methods developed recently can provide presumptive identification of some fungal species (e.g. *C. albicans* or *C. glabrata*) [7], or sensitive simultaneous detection and identification of fungal pathogens directly from blood culture specimens [8]. However, these methods are costly. We sought to establish whether specific molecular components found in *Candida*-positive blood cultures, which we hypothesized would vary according to the pathogen species present, could be exploited to provide rapid species determination by MALDI TOF-MS. This would provide a substantial reduction in the delay before initiation of or adjustment to an effective therapy for invasive candidiasis. Although the costs of MALDI TOF-MS might be considered high at first sight, the costly equipment required is increasingly available in multi-disciplinary laboratories [1], [9]. Moreover, in our laboratory running costs for sample handling and data analysis are slight and lower than those of current biochemical identification methods.

Here, we show that it was possible to identify a *Candida* species by MALDI TOF-MS analysis of an aliquot from the positive blood culture, which removes the need for further lengthy sub-culturing on specific media. This would provide a clinically significant gain of one to two days in the initiation of the most effective therapy.

## Methods

### Experimental and Clinical Samples and Ethics Statement

For blood culture simulation, Mycosis IC/F bottles (Becton-Dickinson, France) were filled with blood of healthy volunteers (Centre de Transfusion Sanguine). Inocula (100 µL) adjusted at 10^4^ yeasts/ml in 0.9% sodium chloride were then inoculated into each bottle (yeast was omitted for control tests). The bottles were then incubated at 35°C with rocking agitation into a Bactec 9240 automat (Becton-Dickinson, France). Growth was continuously and automatically monitored. Simulated blood cultures were replicated to test reproducibility. For each blood culture bottle, two samples were used as biological replicates. 48 *Candida* strains obtained from ATCC or the Pitié-Salpêtrière Hospital (PS) were tested. These belong to 6 species (*C. albicans, C. glabrata, C. krusei, C. lusitaniae, C. parapsilosis* and *C. tropicalis*) representing those most frequently isolated from candidemia (Table 1). The *Candida* strains, stored at −20°C in 10% glycerol, were cultured on CHROMagar medium (Becton-Dickinson, Le Pont de Claix, France) for 48 h at 37°C before being inoculated into blood culture bottles. All the strains tested in this work had been previously identified at the species level using conventional method (API32C biochemical panel, Biomérieux Lyon, France).

**Table 1:** List of the *Candida* species and strains used in the study.

Species	Strains	
	Reference strains	Strains used for validation
*C. albicans*	PS 5441-3, ATCC90028	PS 4667; PS 544; PS 12372; PS 12355; PS 11802; PS 11868, Patient 1: PS 7221
*C. glabrata*	PS 5282, PS 5441-1	PS 569; PS 5282; PS 11188; PS 11189; PS 11594; PS 11597
*C. tropicalis*	PS 5441-2, PS 6325	PS 9359; PS 11185; PS 11186; PS 11187; PS 11745; PS 10597
*C. lusitaniae*	PS 5441-4, PS 6356	PS 5176; PS 9230; PS 922; PS 256; PS 11242; PS 990
*C. parapsilosis*	PS 6, ATCC22019	PS 9360; PS 12338; PS 12339; PS 12340; PS 12341; PS 12342
*C. krusei*	PS 6447, ATCC6258	PS 9395; PS 11190; PS 11191; PS 11192; PS 11193; PS 11194

ATCC, American Type Culture Collection, Manassas, VA, USA. PS, Pitié-Salpétrière Hospital, AP-HP, Paris, France. *used as reference strain for building database in statistical analysis.

Following the development of our method, we had the opportunity to test it on a positive blood culture originating from a kidney transplant recipient who developed lymphoma in 2009 with a worsening condition. Cytotoxic chemotherapy was initiated in June 2009 and the patient was aplastic 6 days later. Due to unexplained fever, the patient was sampled for a set of two blood cultures. One of these was positive after 24 hours of incubation for fungus and *C. albicans* was identified 24 hours later with classical techniques (culture on CHROMagar medium). Sample collection from patient and further analyses were made in full accordance with the tenets of the Declaration of Helsinki. We sought approval for the use of the cultures derived from the blood collected from the patient as part of medical care to the local Institutional Review Board (CPP-Ile-de-France-VI, GH Pitié-Salpêtrière). The IRB trenched that in this particular case, formal IRB approval and written consent from patients are not required (Art. L1121-1 of Public Health code) given that medical care would not be modified by the research process. Simulated blood cultures were carried out with blood obtained within the framework of convention between INSERM and French blood bank (EFS 093031-ABP). According to this procedure, the donor provides a written agreement for the use of donated blood for research purposes.

### Sample Preparation for Proteomic Analysis

Once detected as positive by the Bactec 9240, 2 ml from the blood culture bottle were sampled and centrifuged at 10,000 rpm for 2 min at room temperature. The supernatant was discarded and the pellet washed with 1.5 mL pure water. Following centrifugation, the pellet was re-suspended in 1.5 mL of 0.1% sodium dodecyl sulfate and incubated for 2 min. After centrifugation, the resulting pellet was washed twice in 1.5 mL pure water, centrifuged and suspended in 300 µL of pure water +900 µL of absolute ethanol. After centrifugation, 50 µL of 70% formic acid +50 µL pure acetonitrile were added to the pellet and the subsequent solution was thoroughly vortexed before a final centrifugation and then tested. Because, initiation of methods for identification is sometimes delayed, we also test our method by analysing the profiles obtained following the storage of the positive blood culture bottles for 24 hours at 4°C.

### MALDI-TOF Mass Spectrometry Analysis

Sample preparation for MALDI TOF analysis has been described elsewhere [3]. Briefly, supernatants were distributed on a MALDI AnchorChip slide (Bruker-Daltonics, Bremen, Germany) then air-dried and recovered by α-cyano-4-hydroxy-cinnamic acid (CHCA) matrix (50 mg/ml in 50∶50, acetonitrile:2.5% TFA). DH5a *Escherichia coli* protein extract was used for external calibration.

MALDI-TOF analysis were performed on a Bruker Autoflex I MALDI TOF mass spectrometer with a nitrogen laser (337 nm) operating in linear mode with delayed extraction at 20 kV accelerating voltage. Each spectrum was automatically collected in the positive ion mode as an average of 700 laser shots (50 laser shots at 14 different spot positions). A mass range between 3,000 and 20,000 m/z (ratio mass/charge) was selected to collect the signals with the AutoXecute tool of flexcontrol acquisition software (Version 2.4; Bruker-Daltonics). Only peaks with a signal/noise ratio >3 were considered. Spectra were eligible for further analysis when the peaks had a resolution better than 600. For each bloodculture bottle, we collected mass spectra from 2 biological replicates and 2 technical replicates.

### Data

Pre-processing of the spectra was done using ClinproTool 2.2 software (Bruker Da, Gmbh, Bremen, Germany). Recalibration was performed with base line subtraction. Peak picking (for S/N>4) was performed using calculation of the total average spectrum. Only peaks in the 5000–7400 Da mass range was retained for statistical analysis.

### Statistical Methods

A spectra reference database was built containing seven classes, one for each of the 6 *Candida* species tested plus the negative (yeast-free) control. Each class was defined by the spectra collected from two randomly selected strains from each species (Table 1). This ability to determine the *Candida* species using this reference database was then established using 6 additional strains for each of the six *Candida* species under consideration (i.e. 36 additional strains). Spectra (4 per strain) were then acquired for each of these strains and then compared to the reference database in order to ascribe each to one of the classes defined. The comparison was based on a similarity measure as given by the mean of the Spearman rank correlation coefficients between the spectra from the unknown strain and those of the reference strains used to construct the database: for each class, 8 rank correlation coefficients were computed (between the 2 spectra from the reference database and the 4 spectra from the unknown strain). The mean of these coefficients is indicates the degree of similarity between the unknown strain and each of the reference classes. The species to which the unknown strain is considered to belong is the one for which the best similarity was obtained. The algorithm used to achieve this was implemented in a FORTRAN program written by the authors and will be made available on request.

## Results

We first wished to optimize samples extraction protocols, i.e. to obtain spectra where contaminating blood components are minimized to such an extent that they do not obscure spectral peaks that are specific to fungal cultures. This was carried out using the *C. albicans* susceptible reference strain, ATCC90028. Optimal results were obtained from extraction done on 2 ml of blood culture (detected as positive by the automat) and with SDS 0.1% as detergent.

**Figure 1:**
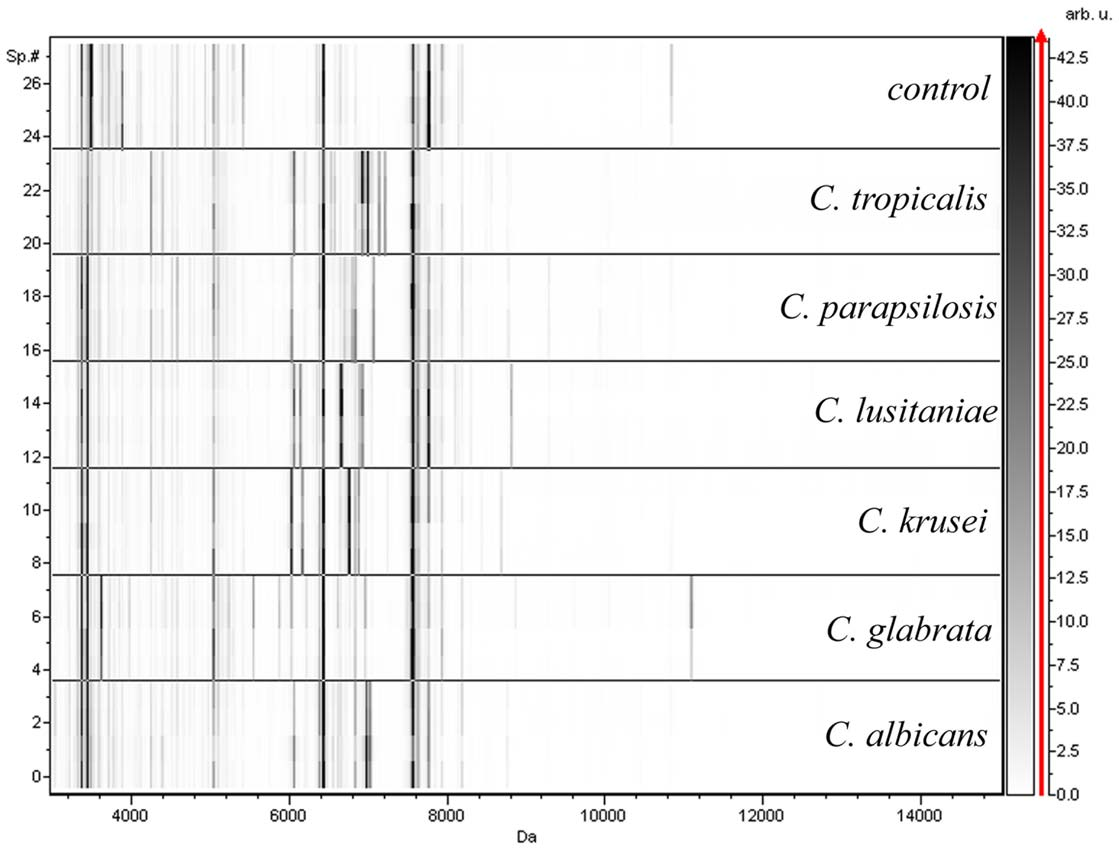
Alterations in the mass spectra (virtual gel) of blood cultures injected with different yeast strains. The x-axis represents Da value, For each strain 4 spectra (numbered on the left Y-axis) from two biological replicates deposited twice on the spectrometer plate are presented. A grey colour scale for peak intensity with arbitrary units is provided on the right y-axis. Each spectrum was automatically collected in the positive ion mode as an average of 700 laser shots. Mass range 3,000–20,000 Da was selected with a signal/noise ratio >3 (S/N) and resolution better than 600 (Flexcontrol software 2.4; Bruker-Daltonics). The control sample was from a yeast-free blood culture.

We then analysed the mass spectrometry fingerprint patterns obtained from aliquots from blood culture bottles inoculated with one or other of two randomly chosen reference strains from each of different *Candida* species: *C. glabrata, C. krusei, C. lusitaniae, C. parapsilosis and C. tropicalis* (Table 1). Typical MALDI TOF-MS spectra obtained from these simulated blood culture are presented in Figure 1. As expected many masses were common to all contaminated and control blood cultures, most probably representing haemoglobins and other blood components. Nonetheless, detectable and quantifiable *Candida*-specific spectral differences (gain or loss of mass) in the 5000–7400 Da mass range were noted between the various yeast-inoculated blood cultures. Some peaks were common to several species, for e.g. the 6903 Da in the spectra of *C. albicans, C. lusitaniae* and *C. tropicalis* blood cultures, while others were specific to each species. These observations were consistently made in reproducibility experiment and for the two strains from the same species. Using these spectra, a reference database was obtained.

In order to ascribe to which species the spectra from a putative clinical sample correspond, we devised a statistical approach based on the mean Spearman rank correlation coefficient used as a similarity measure between groups of spectra. We validated this statistical approach by applying it in fully duplicate experiments (from the inoculation step onwards) to six additional strains (distinct from the reference strains) from each of the six species. As shown in Table 2, the algorithm used led to the correct identification of species of all the 36 additional strains tested. For a subset of the strains (one for each *Candida* species), the positive blood culture had been stored for 24 hours at 4°C before extraction and MALDI TOF-MS analysis.

**Table 2:** Mean correlation coefficients of tested strains and clinical sample of patient against the seven-classes database.

	Class							MS identification	Conventional identification**
Tested strain	*C. albicans*	*C. glabrata*	*C. krusei*	*C. lusitaniae*	*C. parapsilosis*	*C. tropicalis*	Yeast free control		
PS 569	0.312	**0.834**	0.549	0.311	0.379	0.140	0.489	*C. glabrata*	*C. glabrata*
PS 569*	0.366	**0.582**	0.510	0.352	0.422	0.178	0.428	*C. glabrata*	*C. glabrata*
PS 11188	−0.134	**0.571**	0.093	−0.097	−0.003	−0.211	0.181	*C. glabrata*	*C. glabrata*
PS 11189	0.203	**0.724**	0.334	0.244	0.254	0.108	0.348	*C. glabrata*	*C. glabrata*
PS 11594	0.215	**0.552**	0.315	0.332	0.292	0.093	0.226	*C. glabrata*	*C. glabrata*
PS 11597	0.337	**0.659**	0.473	0.442	0.384	0.184	0.445	*C. glabrata*	*C. glabrata*
PS 5282	0.201	**0.809**	0.544	0.293	0.354	0.025	0.520	*C. glabrata*	*C. glabrata*
PS 5176	0.398	0.167	0.357	**0.644**	0.348	0.290	0.247	*C. lusitaniae*	*C. lusitaniae*
PS 5176*	0.395	0.222	0.428	**0.525**	0.407	0.299	0.321	*C. lusitaniae*	*C. lusitaniae*
PS 922	0.415	0.044	0.075	**0.509**	0.083	0.129	0.050	*C. lusitaniae*	*C. lusitaniae*
PS 9230	0.576	0.451	0.475	**0.677**	0.341	0.330	0.550	*C. lusitaniae*	*C. lusitaniae*
PS 256	0.430	−0.047	0.042	**0.536**	0.118	0.181	0.014	*C. lusitaniae*	*C. lusitaniae*
PS 11242	0.526	0.316	0.337	**0.691**	0.291	0.324	0.336	*C. lusitaniae*	*C. lusitaniae*
PS 990	0.378	−0.119	0.026	**0.531**	0.137	0.146	−0.069	*C. lusitaniae*	*C. lusitaniae*
PS4667	**0.751**	0.466	0.502	0.615	0.326	0.401	0.614	*C. albicans*	*C. albicans*
PS4667*	**0.534**	0.103	0.117	0.381	0.082	0.293	0.145	*C. albicans*	*C. albicans*
PS 11802	**0.768**	0.486	0.524	0.730	0.544	0.523	0.566	*C. albicans*	*C. albicans*
PS 11862	**0.722**	0.415	0.354	0.616	0.367	0.391	0.396	*C. albicans*	*C. albicans*
PS 12355	**0.419**	0.022	−0.059	0.252	−0.030	0.238	−0.016	*C. albicans*	*C. albicans*
PS 12372	**0.657**	0.047	0.129	0.449	0.153	0.443	0.222	*C. albicans*	*C. albicans*
PS 5441	**0.506**	0.127	0.128	0.450	0.153	0.287	0.126	*C. albicans*	*C. albicans*
PS 9359	0.401	−0.149	−0.113	0.193	−0.059	**0.615**	0.039	*C. tropicalis*	*C. tropicalis*
PS 9359*	0.418	0.131	0.025	0.310	−0.030	**0.577**	0.103	*C. tropicalis*	*C. tropicalis*
PS 11185	0.460	0.082	0.079	0.350	0.093	**0.674**	0.217	*C. tropicalis*	*C. tropicalis*
PS 11186	0.512	0.011	0.031	0.392	0.056	**0.747**	0.144	*C. tropicalis*	*C. tropicalis*
PS 11187	0.410	−0.104	−0.067	0.263	−0.039	**0.637**	0.050	*C. tropicalis*	*C. tropicalis*
PS 11745	0.112	−0.167	−0.247	0.062	−0.034	**0.261**	−0.080	*C. tropicalis*	*C. tropicalis*
PS 10597	−0.014	−0.543	−0.588	−0.201	−0.314	**0.024**	−0.392	*C. tropicalis*	*C. tropicalis*
PS 9395	0.248	0.622	**0.768**	0.394	0.573	0.034	0.631	*C. krusei*	*C. krusei*
PS 9395*	0.339	0.316	**0.598**	0.416	0.512	0.212	0.365	*C. krusei*	*C. krusei*
PS 11190	0.204	0.106	**0.351**	0.341	0.308	0.088	0.043	*C. krusei*	*C. krusei*
PS 11191	0.075	0.291	**0.427**	0.230	0.350	−0.016	0.180	*C. krusei*	*C. krusei*
PS 11192	0.169	0.390	**0.557**	0.317	0.488	0.083	0.326	*C. krusei*	*C. krusei*
PS11194	−0.033	0.138	**0.309**	0.048	0.308	−0.083	0.098	*C. krusei*	*C. krusei*
PS 11193	0.066	0.352	**0.533**	0.182	0.493	−0.026	0.341	*C. krusei*	*C. krusei*
PS 9360	0.204	0.323	0.626	0.340	**0.657**	−0.030	0.409	*C. parapsilosis*	*C. parapsilosis*
PS 9360*	0.310	0.408	0.678	0.462	**0.751**	0.121	0.459	*C. parapsilosis*	*C. parapsilosis*
PS12338	0.207	0.300	0.654	0.254	**0.760**	0.059	0.435	*C. parapsilosis*	*C. parapsilosis*
PS 12339	0.189	0.328	0.640	0.262	**0.704**	0.028	0.436	*C. parapsilosis*	*C. parapsilosis*
PS 12340	0.168	0.253	0.591	0.216	**0.689**	0.030	0.399	*C. parapsilosis*	*C. parapsilosis*
PS 12341	0.290	0.471	0.707	0.314	**0.727**	0.085	0.635	*C. parapsilosis*	*C. parapsilosis*
PS 12342	0.148	0.309	0.616	0.167	**0.742**	−0.041	0.390	*C. parapsilosis*	*C. parapsilosis*
PS 7221	**0.163**	−0.143	−0.097	0.020	−0.007	0.055	−0.185	*C. albicans*	*C. albicans*

*denotes results obtained from positive blood cultures that had been stored for 24 hours at 4°C prior to SDS extraction. ** See Methods paragraph. The best score is presented in bold.

While we were finalizing the methodology a positive blood culture from a patient admitted to the Pitié-Salpêtrière Hospital became available. MALDI TOF-MS analysis as described above unambiguously identified *C. albicans* as the causal agent, which concords with the identification afforded by routine methods for blood culture.

## Discussion

Candidemia, one of the most common nosocomial bloodstream infection [6], is serious and life-threatening with overall and specific mortality rates of 60 and 49%, respectively, a bleak prognosis that rivals that of bacteraemia [10], [11]. Five species (*C. albicans, C. glabrata, C. parapsilosis, C. tropicalis* and *C. krusei*) account for 90% of cases. Prompt treatment with an appropriate antifungal therapy, which will substantially improves prognosis [12], faces two obstacles. The selection of an effective treatment of candidemia depends on the infecting *Candida* species [6], of which more than twenty each with different antifungals susceptibility profiles [13], [14] are known. At present, species determination requires from 2 to 5 days after collection of the blood samples from the patient.

We present here a proof-of-concept validation of a method based on MS analysis for the accurate and rapid identification of *Candida* species one to two days after collection of the clinical blood sample. This method can be complemented by a MALDI TOF-MS drug susceptibility testing methodology that we have previously developed [15]. The main advantage combining these two assays is the gain of one to two days in the initiation of the most appropriate therapy for patients with a potentially lethal condition.

The genus *Candida* displays extensive genetic diversity and our current database is not exhaustive. We are currently expanding it to include not only other *Candida spp* isolated from human samples but also others yeast (e.g. *Cryptococcus spp*., *Trichosporon spp*.) and filamentous fungi such as *Fusarium spp*. We have initiated an evaluation of MALDI TOF-MS fungus identification in a clinical setting in order to establish its potential to substantially improve the management of candidiasis through very early administration of the most appropriate antifungal therapy. This will help to reduce costs as well as to minimize the selection of drug-resistant fungal pathogens.
